# Effect of Basic Amino Acids on Folic Acid Solubility

**DOI:** 10.3390/pharmaceutics15112544

**Published:** 2023-10-27

**Authors:** Karen Pérez-Carreón, Luz María Martínez, Marcelo Videa, Jorge Cruz-Angeles, Jimena Gómez, Emilio Ramírez

**Affiliations:** School of Engineering and Sciences, Tecnologico de Monterrey, Campus Monterrey, Ave. Eugenio Garza Sada 2501 Sur. Monterrey N.L., Monterrey 64849, Mexico

**Keywords:** folic acid, amino acids, arginine, lysine, solubility, ball milling, amorphous drug, conductimetry

## Abstract

To prevent neural tube defects and other cardiovascular diseases in newborns, folic acid (FA) is recommended in pregnant women. A daily dose of 600 µg FA consumption is widely prescribed for women during pregnancy and 400 µg for women with childbearing potential. FA is a class IV compound according to the Biopharmaceutics Classification System (BCS) due to its low permeability (1.7 × 10^−6^ cm/s) and low solubility (1.6 mg/L); therefore, it must be administered via a formulation that enhances its solubility. Studies reported in the literature have proved that co-amorphization and salt formation of a poorly soluble drug with amino acids (AA) can significantly increase its solubility. Although arginine has been used with FA as a supplement, there is no information on the effect of basic AA (arginine and lysine) on the physical and chemical properties of FA-AA binary formulations. The present study implemented a conductimetric titration methodology to find the effective molar ratio to maximize FA solubility. The results showed that a 1:2.5 FA:AA molar ratio maximized solubility for arginine and lysine. Binary formulations were prepared using different methods, which led to an amorphous system confirmed by the presence of a glass transition, broad FTIR bands, and the absence of an X-ray diffraction pattern. Results of FA:AA (1:2.5) solubility increased in the range of 5500–6000 times compared with pure FA. In addition to solubility enhancement, the binary systems presented morphological properties that depend on the preparation method and whose consideration could be strategic for scaling purposes.

## 1. Introduction

Folic acid (FA), also known as vitamin B9, is necessary for the healthy functioning of various processes occurring in the body, such as DNA synthesis, DNA repair processes, catecholamine and pyrimidine synthesis, purine synthesis, and the conversion of homocysteine to methionine [1,2,3,4,5]. Conversely, folic acid deficiency is associated with conditions such as megaloblastic anemia, elevated homocysteine levels, cardiovascular diseases, and neural tube defects in fetuses (e.g., anencephaly or spina bifida) [6,7]. In the gestational period, FA promotes the fetus’s growth and development, expands the placenta’s blood vessels, and increases blood supply [8,9]. However, the human body cannot produce this substance. Therefore, the recommended dietary intake (RDA) for folate is 400 µg/day in adults and 600 µg/day in pregnant women [10,11]. According to an extensive review by Czeizel et al. [12], periconceptional supplementation can prevent neuronal tube malformations in newborns by 70%. To meet the demand for these substances, pregnant women must consume this particular nutrient to prevent malformation of nervous system organs in the fetus and contribute to the normal homocysteine metabolism.

**Figure 1:**
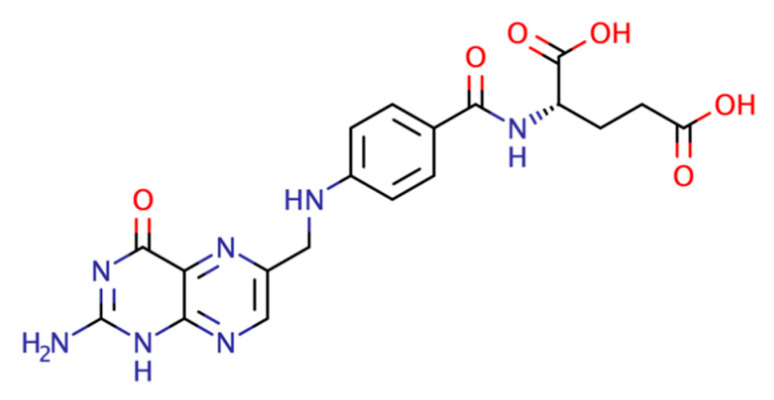
Folic acid structure.

FA is an active pharmaceutical ingredient (API) classified as a class IV compound according to the BCS due to its low permeability (1.7 × 10^−6^ cm/s) [13] and low solubility (1.6 mg/L) [14] (see Figure 1). The common approach to increase its solubility is to prescribe the sodium salt form. However, sodium intake formulations should be limited in people with hypertension since 5% to 10% of pregnancies worldwide are complicated by hypertensive disorders [15]. Sodium-free formulations are desirable to increase the solubility of FA. Moreover, poorly water-soluble drugs often require high doses to reach therapeutic plasma concentration after oral administration [16].

Most commercially available tablet formulations stumble with the dissolution of FA since these exhibit different dissolution rates and low dissolution levels, failing to meet USP (US Pharmacopeia) requirements; this severely affects the therapeutic dose needed for specific supplementation required (e.g., pregnant women with NTDs history) [17,18]. Therefore, the search for methodologies to enhance solubility and permeability is highly relevant.

There are several strategies to enhance the solubility of an active pharmaceutic ingredient (API) with an acidic nature, such as FA, one of which is using a co-former with a basic character to form a salt with a better interaction with water and thus enhancing its solubility. Salt systems are composed of an ionizable acid or donor (base or acceptor) API and a conjugate co-former, which results in an adduct with cationic and anionic characteristics [19,20,21,22]. Salt formation has become an essential step in pharmaceutical development, and it is widely used in the industry to improve the solubility and dissolution rate of poorly soluble drugs [23].

Based on the arrangement of the molecules or ions, either a crystalline or an amorphous system may form [23]. A crystalline structure will have a well-defined distribution of the molecules in a crystalline lattice, while in an amorphous structure, molecules present a short-range disorder. Amorphization is the transformation of an API from its crystalline form into its amorphous state, where crystalline order is absent. In this state, solubility improvement is observed, given that structurally disordered materials have higher free energy than their crystalline form, increasing the spontaneity of their dissolution [24,25,26,27,28,29,30,31,32]. Salt formation and amorphization are good alternatives to achieving solubility enhancement of poorly soluble drugs [28]. Furthermore, the use of amino acids (AA) as co-formers has been studied and found to be reliable as they have low molecular weight, are common constituents of the daily diet, and are non-toxic [33,34]. Also, the variety of functional groups in their side chains may lead to a wide range of interactions that could result in a formulation with an API’s desired stability and high solubility [35].

**Figure 2:**
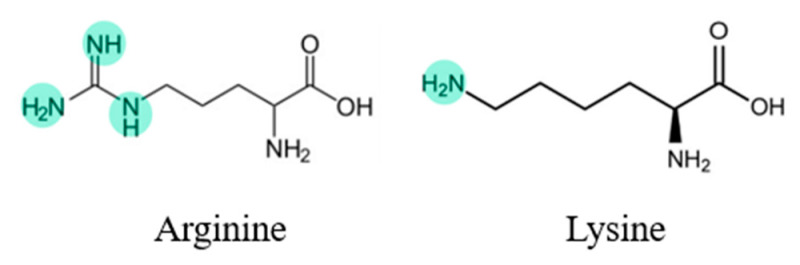
Arginine and lysine structures.

Acid–base interactions may lead to solubilities exceeding the pure low-solubility APIs [36]. Various studies have reported the enhancement achieved with formulations of acid-APIs combined with basic AA, such as arginine (ARG) and lysine (LYS), which have amino groups in their side chain (see Figure 2) [34]. These groups provide a basic reactivity toward the acidic functional groups of the selected API to form a salt. Regarding the formation of salts, according to Shemchuk et al. [36], a difference of 2 or more between the *pK*_b_ (base) and *pK*_a_ (acid) values may determine salt formation in binary formulations since the probability of a proton transfer is high, and salt formation is expected [20,36].

Table 1 presents examples of basic amino acids used in binary formulations with acidic APIs. An increase in APIs’ solubility is observed when combined with a basic amino acid co-former, even in the case of a physical mixture. For example, in the physical mixture of indomethacin–arginine, a 3.4-fold increase in the solubility of pure crystalline indomethacin was reported, only 20% below the increase achieved in the co-amorphous mixture.

Although formulations with AA have been studied at equimolar ratios [37,38,39], the molar ratio between the APIs and the co-formers is an essential parameter in a formulation, and Liu et al. [40] determined that equimolar compositions only sometimes maximize solubility since many interactions may exist between components. Different studies have reported that solubility enhancement of folic acid can be maximized at molar ratios of FA: Co-former of 1:2 [4,41].

Because of their acidic nature, basic AAs are suitable co-formers for obtaining a formulation with higher solubility. However, some studies include a combination of arginine with FA as supplements [42,43,44,45,46,47] and histidine as a solubility enhancer if liposome encapsulated FA [2], although the authors do not mention the proportion of folic acid and histidine. To the best of the authors’ knowledge, there are no previous studies of binary systems of FA with basic amino acids formulated to maximize its solubility and to study the effect of its preparation methods on the formulation’s physicochemical properties. This paper aims to explore these binary systems using ARG and LYS as co-formers and to determine the ratio between folic acid and amino acid of the highest solubility through the implementation of a conductimetric titration, which has not been used for this kind of acid–base binary amorphous drug formulation. Moreover, enhancing the solubility and bioavailability of folic acid in combination with other essential nutrients may lead to multicomponent therapeutic and nutritional formulations with expectedly higher solubility, and thus higher bioavailability, than current formulations for preventing and treating chronic diseases during pregnancy.

## 2. Materials and Methods

The following reagents were acquired and used without any further treatment. Folic acid (FA), (C_19_H_19_N_7_O_6_, MW = 441.4 g/mol Sigma Aldrich, St. Louis, MO, USA), L-Lysine (LYS) (C_6_H_14_N_2_O_2_, ≥98%, MW = 146.19 g/mol, Sigma Aldrich, St. Louis, MO, USA Lot. 13CCB3586), L-Arginine (ARG) (C_6_H_14_N_4_O_2_, ≥98%, MW = 174.20 g/mol, Sigma Aldrich, St. Louis, MO, USA, Lot. 700640615). MiliQ-Water was used as the solvent (18.2 mΩ), Glacial acetic acid (CH_3_COOH, 100%, MW = 60.05 g/mol, J.T. Baker, Lot. K25C70), Acetonitrile (ACN, HPLC grade MW = 44 g/mol Honeywell, St. Harvey, MI, USA).

### 2.1. Conductimetric and Potentiometric Titration of Folic Acid with Amino Acid for Composition-Solubility Optimization

Conductometric and potentiometric titrations were conducted to establish the effective molar ratio at which the full acid–base interaction of FA with the AA occurred. Conductivity and *p*H measurements were carried out while adding 10 μL aliquots of an aqueous solution of AA of a known concentration to 10 mg of crystalline powder FA suspended in 10 mL of deionized water. A Vernier conductivity meter and potentiometer were used, and the data was collected with Logger Pro software version 3.8.4.

### 2.2. Preparation of Binary Formulations of FA:AA

Three methodologies of preparing the binary formulation of FA:AA were explored with the molar ratio of 1:2.5 found in the conductimetric titration experiments.

(1) Physical mixture (**CPM**), mixing the two pure crystalline components without further process;
(2) Amorphous salt formation by solvent evaporation (**ASE**) in which a physical mixture of the two pure crystalline components was dissolved in water and then dried under a vacuum oven overnight.

The FA:AA ASE binary systems were prepared in a 5 mL beaker weighing a total mass of 100 mg of sample and 1 mL of MiliQ-Water. The solution was stirred for approximately 50 min and covered to prevent light exposure. The solution was dried under a vacuum oven at a temperature of 60 °C. A small sample was collected and analyzed using a Simultaneous Thermal Analyzer STA 6000 (PerkinElmer^®^, Waltham, MA, USA). 10 mg of the sample were heated from 37 °C to 110 °C at a heating rate of 10 °C/min and held isothermally to record any weight loss for an hour.

(3) Amorphous by ball milling (**BM**), in which the two pure crystalline components were intimately mixed to obtain a fine powder.

The FA:AA BM binary system was prepared, weighing a total mass of 100 mg. The system was amorphized using a Fritsch Pulverisette 7 planetary ball mill. The binary system was placed inside a stainless-steel grinding bowl with six 10 mm stainless steel balls and ground at 850 rpm at 15 min intervals with 10 min rests to prevent degradation of FA through heat until amorphization.

### 2.3. Structural Characterization by Powder X-ray Diffraction (PXRD)

To evaluate the crystallinity of the samples, structural analysis was performed using an X-ray diffractometer, Miniflex 600, Rigaku (Tokyo, Japan), and a Cu (Kα) cathode as the source. The conditions used in all analyses were a voltage of 40 kV and a current of 15 mA; a 3° to 40° was measured with a step of 0.05° and a speed of 2°/min. Samples were recovered and stored in a desiccator under a vacuum after analysis.

### 2.4. Intermolecular Interactions by Fourier-Transform Infrared Spectroscopy (FTIR)

To characterize the intermolecular interactions between FA and AA, binary formulations and pure components were analyzed using a Perkin Elmer FTIR equipment, Spectrum 100 model. All spectra were acquired with a resolution of 4 cm^−1^ in a range from 4000 to 380 cm^−1^. For the analysis of the spectra, Perkin Elmer spectrum software version 10.6.0 was used.

### 2.5. Solubility Measurements

The solubility of FA was determined by reversed-phase high-pressure liquid chromatography (HPLC). An Agilent Series 1100 (Agilent Technologies, Santa Clara, CA, USA), equipped with a G1322A degasser, a G1311A quatpump, a G1313A autosampler (ALS) and a G1314A UV-VIS detector, was used. Moreover, 20 µL samples were injected onto a 150 mm × 4.60 mm hypersil gold C_18_ selectivity 5 µm protected by a pre-column guard cartridge (Thermo Scientific, Rochester, NY, USA).

For the analysis of FA and its salts, a mobile phase composed of 0.1% acetic acid and acetonitrile (ACN) in a ratio of 86:14 previously filtered through a 0.2 µm, 47 mm diameter nylon membrane (Thermo Scientific, Dreieich, Germany), was used. The isocratic flow rate was set at 1.4 mL/min, the column temperature was maintained at 25 °C, and the detection wavelength was 280 nm. The methodology was based on Osseyi et al. [48].

A standard solution of FA was prepared to construct a reliable calibration curve. Given the low solubility of FA in water, ARG was added to create a stock solution at molar ratio FA: ARG of 1:2 and a concentration of 50 ppm. From three stock solutions, dilutions ranging from 0.5 to 50 ppm were prepared with 14 intermediate points. These were used to determine linearity and construct calibration curves. Since FA is light sensitive [49,50], all solutions were stored at 4 °C and protected from light exposure. Each solution was evaluated in triplicate and filtered through a 0.2 µm syringe filter before analysis.

To address the significant differences in solubility between pure FA and binary formulations, two different experimental approaches were employed, both designed to ensure the presence of excess FA. In the first approach, 5 mg of FA binary formulation was taken and mixed in an Eppendorf tube with 20 µL of water. The samples were centrifuged for 3 min at 5000 rpm, then subjected to four iterations of vortex at 3000 rpm for 3 min and centrifugation at 10,000 rpm for 5 min. Once complete dissolution was confirmed, the sample underwent final centrifugation at 10,000 rpm for 5 min to prepare for analysis. The samples underwent consecutive dilutions before injecting 5 μL of the final solution into the HPLC column. The samples underwent consecutive dilutions before injecting 5 μL of the final solution into the HPLC column. In the second approach, 4 mg of pure FA was dissolved in 50 mL of water and stirred for 24 h at room temperature under dark conditions to prevent photodegradation. With the first approach, a theoretical maximum concentration of 125,000 ppm FA would be reached, whereas with the second approach, the concentration would be 80 ppm. Regardless of the approach, all samples were filtered using a 0.2 µm pore size syringe filter and subjected to triplicate analysis by HPLC.

### 2.6. Thermal Stability of the Formulations by Simultaneous Thermal Analysis

The different FA formulations were analyzed in a Perkin Elmer Simultaneous Thermal Analyzer STA 6000 (PerkinElmer^®^, Waltham, MA, USA). Measurements were performed in 25 μL ceramic sample pans containing around 10 mg of sample. The samples were heated from 15 °C to 140 °C at a heating rate of 10 °C/min under a nitrogen atmosphere. Before use, the system was purged with nitrogen (19.8 mL/min) for one hour. The instrument was calibrated using metallic Indium, corroborating its melting temperature at 156.6 °C.

### 2.7. SEM Inspection of the Binary System’s Morphology

To characterize the morphology of the binary systems produced, samples were prepared by casting the powders of the physical mixture of LYS and ARG with FA and the systems prepared by solvent evaporation (ASE) and ball milling (BM) on a carbon adhesive tape attached to an aluminum sample holder. The samples were inspected in a Phenom Pro X scanning electron microscope operated at 5 kV with a backscattering detector.

## 3. Results

### 3.1. Conductimetric and Potentiometric Titrations

**Figure 3:**
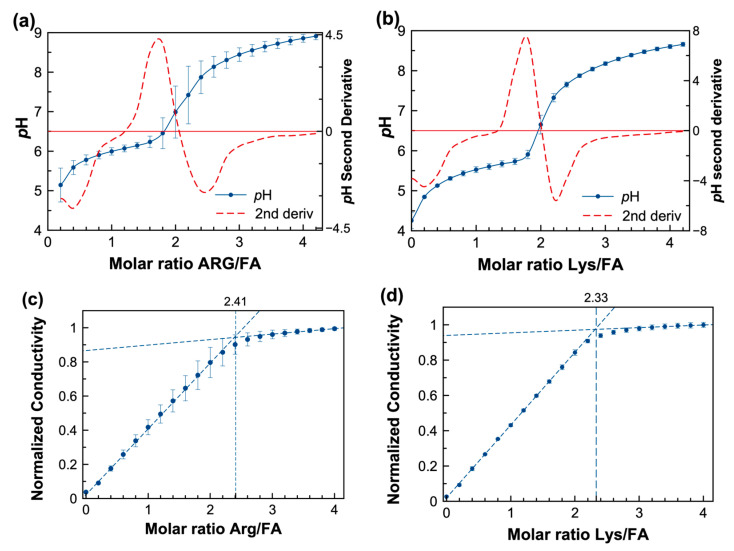
(**a**,**b**) potentiometric titration of FA with lysine (Lys) and arginine (Arg) and numerical second derivative of *p*H (red dashed line), respectively (**c**,**d**) conductimetric titration of FA with a concentrated solution of ARG and LYS, respectively; the extrapolation dashed lines cross at the molar ratio of maximum conductivity.

In this experiment, the acid–base reaction between FA and lysine (LYS) and arginine (ARG) was monitored during a potentiometric titration, where an increase in the *p*H followed the addition of the amino acid, expressed as molar ratio, as shown in Figure 3a,b. The FA:AA system acts as a buffer in the 4.5–6.0 *p*H region. Several studies have reported that the solubility of folic acid is pH-dependent [8,51,52]. Additionally, the second derivate demonstrates (red dashed lines in Figure 3a,b) the change of slope around the equivalent molar ratio of FA:AA of 1:2 for both amino acids.

Conductivity was measured in similar titrations since it represents a convenient tool for studying systems in which ionic species are produced due to the acid–base reaction between FA and basic amino acids such as LYS or ARG [53]. In the conductimetric titration curves (Figure 3c,d), the conductivity increased linearly when LYS or ARG were added to the FA. Then, maximum ionization occurs when the conductivity no longer increases, as shown by the intersecting slopes. When extrapolating both regions of the conductivity, a crossover occurs at an FA:AA molar ratio of 1:2.33 for FA:LYS and 1:2.41 for FA/ARG, respectively. Macfie et al. refer to this intercept as the conductivity point corresponding to a saturated solution [54]. The conductimetric titrations suggest that a maximum solubility for the FA:AA systems is reached at a molar ratio of approximately 1:2.5. Thus, the solubility enhancement achieved at this molar ratio was explored for both systems.

### 3.2. Structural Characterization by X-ray Diffraction (XRD)

**Figure 4:**
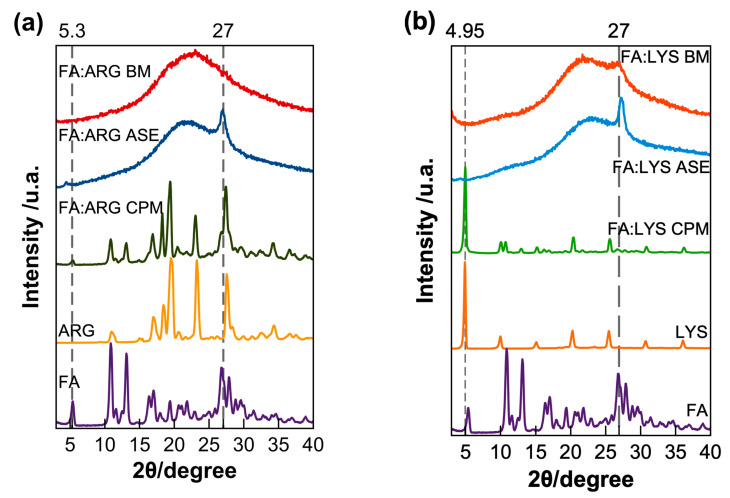
X-ray diffraction of pure components and formulations of (**a**) arginine and (**b**) lysine pure components along with the physical mixtures (CPM), amorphous by solvent evaporation (ASE), and the ball milled (BM). For comparative purposes, each diffraction pattern is normalized with respect to its maximum intensity.

Figure 4 compares the diffraction patterns for the different FA formulations. It can be noticed that the diffraction pattern for the simple physical mixture (CPM) is equivalent to the sum of signals of each pure component. For the FA:AA-ASE formulation, a broad halo, characteristic of amorphous materials, is observed, except for a peak at 27° for both lysine and arginine (Figure 4). In the case of the ball milling formulations FA:ARG-BM, a complete amorphization was achieved after eight ball milling cycles of 15 min, while the formulation FA:LYS-BM was only amorphized after 14 cycles of 15 min but showed a broad peak at 27°, as observed in the systems prepared by solvent evaporation (ASE). The presence of this diffraction peak was previously observed, and it is interpreted, according to Magri et al., as a result of the self-assembly of dianionic folate ions through hydrogen bonds and π−π stacking [55].

### 3.3. Intermolecular Interactions Results

The IR spectra of pure components and all binary samples were acquired to evaluate the presence of molecular interactions due to the formation of salts. In the case of pure FA, the band at 1696 cm^−1^ is attributed to the stretching vibration of the C=O of carboxylic acid and, at 1604 cm^−1^, is related to the bending of the N-H group. Moreover, in 1680 cm^−1^, the band is associated with the phenyl ring; finally, the peak in 1409 cm^−1^ is associated with the deformation of the O-H bond in the phenyl skeleton and, in 2928 cm^−1^, is attributed to the C-H bond [56]. According to Kasten et al., the antisymmetric stretch of the corresponding ionized carboxyl group is found between 1505 and 1610 cm^−1^ [23]. For example, the study of Magri et al. details how the formation of sodium folate salts affects the structure of the molecule and, consequently, its vibrational spectrum, especially the signals associated with the carboxyl groups, which are displaced towards lower energy ranges in the spectra of folate salts compared to folic acid, suggesting intermolecular hydrogen bonding and weakening of the C=O bond. Furthermore, some bands present in the spectra of folic acid are absent in those of folate salts, indicating deprotonation of the glutamic acid moiety and formation of an enolate group. This absence of bands and the displacement observed in others are key evidence for the formation of folate salts [55]. Thus, this spectral region was selected for analysis.

**Figure 5:**
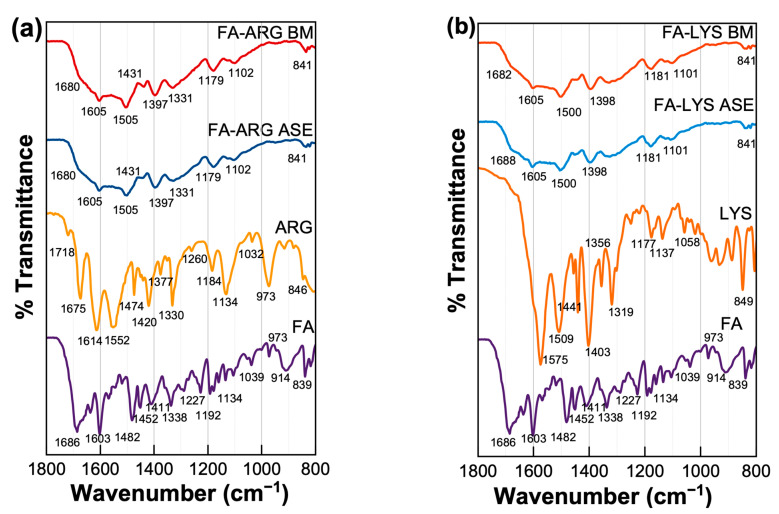
Infrared spectra of different FA formulations and pure components, (**a**) the FA:LYS systems and (**b**) the FA: ARG systems, are shown and obtained with Perkin Elmer spectrometer model Spectrum 100.

In addition to pure folic acid, Figure 5 also shows the spectra of the FA-LYS and FA-ARG formulations. In both formulations, under the two types of preparation, the signals’ broadening is evident, reflecting the amorphization of the samples. Following the study by Magri et al. [55], the movement or absence of carboxyl group signals was analyzed, which could be interpreted as deprotonation and possible formation of a salt. When comparing the signals of 1686 cm^−1^ of the carboxyl of pure folic acid with the FA:AA formulations, we can observe that there is a decrease in signal intensity, and, in some cases, these signals are absent. These changes could be interpreted as the possible formation of a salt in both cases.

### 3.4. Solubility Results

**Figure 6:**
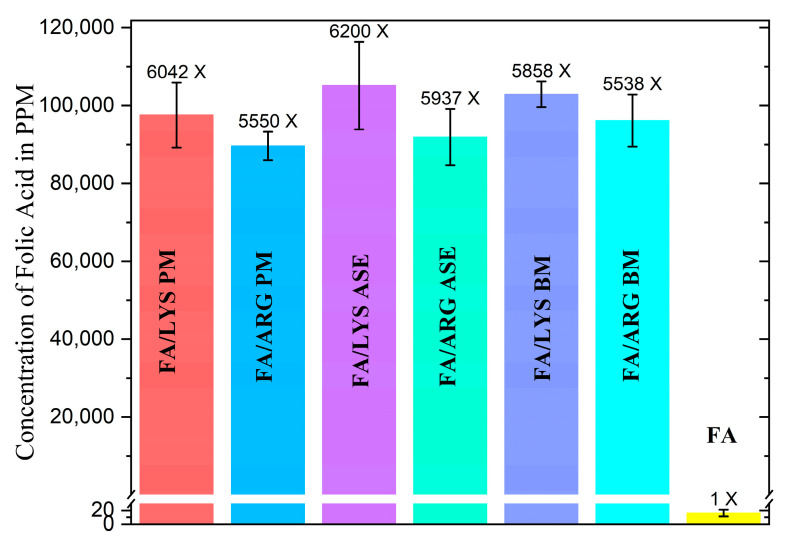
Comparison of solubilities in water of different formulations, with error bars (*n* = 3), the increment of solubility in comparison with FA alone is above bars. A one-way ANOVA analysis demonstrated that the difference between all media of all FA formulations was not significant with a *p*-value of 0.014; no matter the different changes in their crystal structure, the solubility of FA has proven not to change significantly. This could be attributed to other unseen root causes.

Figure 6 shows the solubility of FA and the different formulations at the molar relation of FA:AA of 1:2.5. FA solubility was found to be 16.15 mg/L, closely related to the value reported in the literature of approximately 10 mg/L [14,57]. The addition of ARG and LYS remarkably improves FA’s solubility at least 6000 times, regardless of the preparation method, which could be attributed to the formation of folate salts, resulting in greater solubility performance due to electrostatic acid–base interactions present [23,58].

### 3.5. Thermal Properties of the FA Formulations

**Figure 7:**
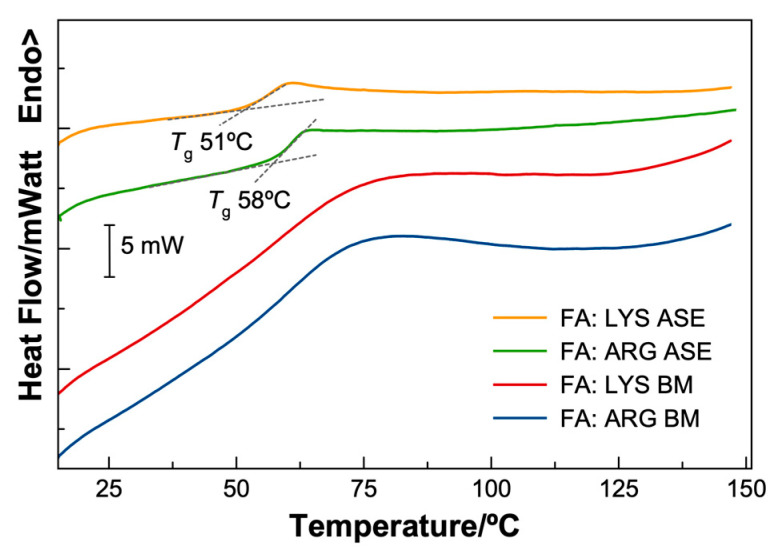
Thermograms for the FA formulations. The solvent evaporation formulations produce amorphous materials with defined onset glass transitions: 51 °C for FA-LYS ASE and 58 °C for the FA-ARG ASE. In the case of the ball-milled formulations, FA-LYS BM and FA-ARG BM, no clear onset can be determined.

The thermal properties of the FA formulations were evaluated from 20 °C to 150 °C at a 10 °C/min heating rate, using simultaneous thermal analysis, i.e., differential thermal analysis (DTA) and thermogravimetric analysis (TG). The DTA thermograms for the ASE and BM systems are shown in Figure 7. FA-LYS ASE and FA-ARG ASE prepared by solvent evaporation present distinct glass transition steps with onsets at 51 °C and 58 °C, respectively, confirming their amorphous structure. On the other hand, the thermograms corresponding to the ball-milled formulations show a broad peak that extends from 50 °C to 75 °C. No crystallization processes are observed after the glass transition when heating up to 150 °C.

### 3.6. Morphological Inspection of the FA-AA Systems

**Figure 8:**
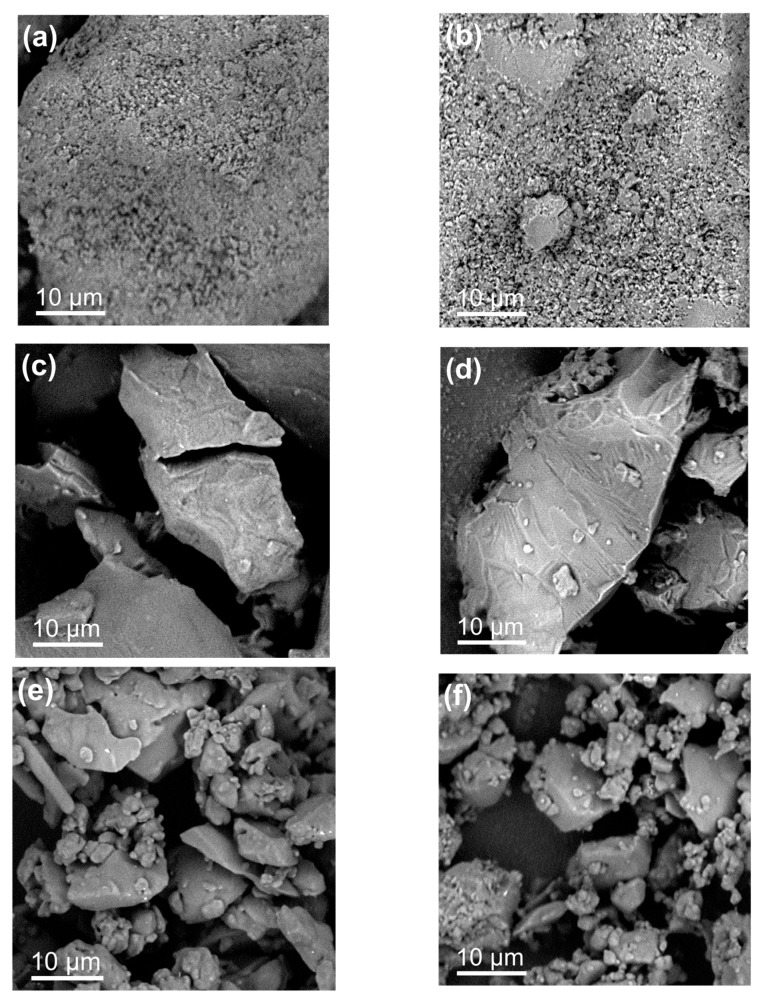
SEM images of the crystalline physical mixture (CPM) of (**a**) FA-LYS and (**b**) FA-ARG, amorphous salt by solvent evaporation (AES), (**c**) FA-LYS and (**d**) FA-ARG, and amorphous by ball milling (BM) (**e**) FA-LYS and (**f**) FA-ARG.

The morphology of the powders obtained from the different formulations was inspected under electron microscopy and shown in Figure 8. The physical mixtures of the crystalline FA and Lys (Figure 8a) and FA and Arg (Figure 8b) produce aggregates of particles of the order of 1 um. In the case of the binary systems obtained from solvent evaporation, both for FA-Lys (Figure 8c) and FA-Arg (Figure 8d), compact particles of the order of tens of micrometers are observed whose surfaces present typical fracture patterns of amorphous materials, consistent with the X-ray diffraction results, confirming that the binary systems amorphized as a result of the drying process. A similar morphology of amorphous particles is observed in the case of the binary systems obtained by ball milling, although in this case, the particle size is of the order or smaller than 10 um FA-Lys (Figure 8e) and FA-Arg (Figure 8f).

## 4. Conclusions

The present study implemented a conductimetric titration method to determine the FA:AA molar ratio that maximizes folic acid’s solubility. Finding the optimal required amount of the amino acids prevents administration in an excess dose that could otherwise be disadvantageous. Although conductimetric titration is well known for monitoring acid–base reactions, to our knowledge, the method has not been used to measure the acid–base interaction between folic acid and a basic solubility enhancer. The technique allows the measurement of soluble charge-carrying species, which directly correlates to the solubility of the folic acid. Thus, the conductimetric technique allows for determining the composition of the highest solubility of folic acid with an amino acid, which does not necessarily coincide with a stoichiometric ratio. As a result, it was found that this molar ratio was close to 1:2.5, leading to an increase in FA solubility of at least 6000 times for both basic amino acids ARG and LYS compared to pure FA, regardless of the formulation method. These amino acids were proved to be good co-formers for amorphous folate salt formation with glass transitions well above room temperature. Amorphization through ball milling produced smaller particles than those obtained from solvent evaporation. This work contributes an effective means of administering a highly soluble folic acid formulation.

## Figures and Tables

**Table 1:** Examples of binary formulations of acidic APIs and the basic AA arginine.

API	Amino Acid	Formulation Type	Molar Ratio	Solubility Fold Increase	Ref.
Indomethacin	Arginine	Co-amorphous Physical mixture	1:1	4.10 3.40	[37]
Ciprofloxacin	Arginine	Amorphous solid dispersion Physical mixture	1:1	12.0 7.0	[38]

## Data Availability

The data supporting this study’s findings are available from the corresponding author upon request.
